# Octopamine Signaling Regulates Phototactic Behavior via a β-Adrenergic-like Receptor in *Diaphorina citri*

**DOI:** 10.3390/insects17080851

**Published:** 2026-08-15

**Authors:** Fei-Feng Wang, Xiu-Qi Zou, Ming Zhong, Sheng-Feng Tu, Min-Er Li, Wen-Feng Zhao, Bao-Li Qiu, Wen Sang

**Affiliations:** 1 Engineering Research Center of Biotechnology for Active Substances, Ministry of Education, Chongqing Normal University, Chongqing 401331, China; wff1013634299@163.com; 2 Engineering Research Center of Biological Control, Ministry of Education, South China Agricultural University, Guangzhou 510640, Chinazwf555@scau.edu.cn (W.-F.Z.)

**Keywords:** octopamine, tyramine beta-hydroxylase, octopamine receptor beta-1R, *Diaphorina citri*, pharmacology, phototaxis

## Abstract

The Asian citrus psyllid (*Diaphorina citri*, ACP) is the primary vector of citrus huanglongbing and exhibits positive phototactic behavior. Octopamine (OA) is a critical neuromodulator of diverse insect behaviors, yet its regulatory mechanism in ACP phototactic behavior remains largely uncharacterized. In this study, three core genes of the OA signaling pathway in ACP were identified and characterized: the biosynthetic enzyme *DcTβH* and two β-adrenergic-like OA receptors (*DcOctβ1R*, *DcOctβ2R*). All three genes exhibited a U-shaped developmental expression pattern and high transcript abundance in the head, suggesting potential involvement in the early nymphal and adult stages and essential roles in central nervous system. Pharmacology assays revealed that DcOctβ1R activation by OA and tyramine induced cyclic adenosine monophosphate (cAMP) accumulation, with agonists and antagonists exerting differential effects on its activity. In contrast, no cAMP response was observed in cells expressing *Dc*Octβ2R regardless of ligand treatment. Combined RNA interference and behavioral assays demonstrated that silencing *DcTβH* or *DcOctβ1R* significantly impairs ACP phototactic behavior. These findings revealed that the OA signaling positively regulates phototactic behavior via *DcOctβ1R* in ACP. This study enhances the understanding of OA-mediated phototactic behavior in agricultural pests and lays a foundation for exploring the molecular mechanisms of insect behaviors.

## Simple Summary

The Asian citrus psyllid (*Diaphorina citri*) is the primary vector of citrus huanglongbing, a destructive disease that causes severe losses to citrus production worldwide. *D. citri* exhibits positive phototaxis, the innate tendency to move toward light. However, the molecular mechanisms that control this key behavioral trait remain poorly understood. In this study, we investigated the role of octopamine, a core neuromodulator that governs multiple behaviors in insects, in regulating *D. citri* phototactic behavior. We identified three key genes in the octopamine signaling pathway, analyzed their expression patterns, characterized the pharmacological response of receptors, and validated their behavioral functions via RNA interference. Our results show that the octopamine positively regulates phototactic behavior via *DcOctβ1R* in *D. citri*. These findings deepen our understanding of the molecular basis of insect behavior.

## 1. Introduction

Phototactic behavior, a prevalent and ecologically important trait across insects, shapes critical ecological interactions such as habitat selection and reproductive behaviors [1,2,3]. The internal mechanisms of phototaxis have been well studied in *Drosophila melanogaster* [4,5,6]. Although phototaxis has been studied in various taxa, most studies have only focused on behavioral and physiological aspects, such as preferred light wavelength and intensity [7,8,9]. The Asian citrus psyllid (ACP), *Diaphorina citri* (Hemiptera: Liviidae), is a major pest threatening global citrus production and the primary vector of citrus huanglongbing (HLB). Systematic behavioral assays further demonstrated that ACP exhibits positive phototaxis and preferentially responds to light wavelengths of 390–400 nm and 520–525 nm. In addition, the phototactic attraction is intensity-dependent in ACP, with higher irradiance eliciting stronger phototactic responses [10,11,12]. These behavioral characteristics have laid an empirical foundation for light-trapping technology targeting ACP, yet the molecular and neurobiological basis underlying its phototactic response remains largely uncharacterized.

Octopamine (*p*-hydroxyphenylethanolamine, OA) was first detected in the venom-producing posterior salivary gland of *Octopus vulgaris* [13]. As an important biogenic monoamine in invertebrates, OA is highly concentrated in insect neurons, whereas it exists only as a trace amine in vertebrates. Structurally similar to noradrenaline, OA acts as a neurohormone, a neuromodulator and a neurotransmitter in invertebrates [14]. The known actions of OA in the central nervous system include desensitization of sensory inputs, influence on learning and memory, or regulation of the “mood” of the animal [15,16,17]. Moreover, accumulating evidence from diverse insect models demonstrates that OA signaling serves as a pivotal regulator of diverse behavioral processes, including reproductive output [18], foraging and feeding [19], aggressive behavior [20], flight [21] and locomotion [22]. Synthesized from tyramine via hydroxylation by tyramine β-hydroxylase (TβH) [23], OA exerts its functions by binding to OA receptors. OA receptors belong to the G protein-coupled receptor (GPCR) family and mediate intracellular signaling by modulating the levels of second messengers, including cyclic adenosine monophosphate (cAMP) and/or intracellular calcium [24]. OA receptors are classified into three major groups: α-adrenergic-like OA receptors (OctαRs), β-adrenergic-like OA receptors (OctβRs), and OA/tyramine receptors [24]. OA modulates behavior through the cooperative function of diverse receptor subtypes. In *Nicrophorus vespilloides*, for example, *Octβ1R* and *Octβ2R* are upregulated by mating and downregulated by reproductive resources, whereas *Octβ3R* shows no mating response and is induced by either a mate or resources alone. *OctαR*, in contrast, is downregulated solely under the combined condition of both mates and reproductive resources [25]. Functional studies have further demonstrated that OctαR mediates reproduction and associative learning, while OctβRs are primarily involved in locomotion, reproduction, and stress resistance [22,26,27,28]. The subtype-specific functional divergence of OA receptors provides a molecular basis for the precise modulation of environmentally guided behavioral responses in insects. Notably, OA titers were found to control phototactic behavior in honeybees [29,30]. Despite these findings, the regulatory functions and underlying mechanisms of key genes in the OA signaling pathway in regulating phototactic behavior are still mostly uncharacterized, especially in major pests that are sensitive to light and color.

In ACP, our previous transcriptomic analysis between phototactic and non-phototactic individuals revealed significant differences in the expression levels of Octβ1R between the two phenotypic groups [31], suggesting that the OA signaling pathway may participate in modulating the phototactic behavior of ACP. However, the causal regulatory roles of functional genes in the OA pathway in this behavioral process have not been experimentally validated. In this study, we focused on key genes in the OA signaling pathway and investigated their effects on the phototactic behavior of ACP. Three key genes involved in OA signaling pathway were identified in *D. citri*: the OA biosynthetic enzyme *DcTβH* and two β-adrenergic-like receptors, *DcOctβ1R* and *DcOctβ2R*. All three genes exhibited a U-shaped developmental expression pattern and high transcript abundance in the head. Pharmacological assays revealed that OA and TA induced cAMP accumulation via DcOctβ1R, which also exhibited differential potency against the tested agonists and antagonists. Additionally, combined RNA interference (RNAi) and behavioral assays demonstrated that OA signaling positively regulates phototactic behavior via DcOctβ1R in ACP. Collectively, these findings advance our understanding of OA-mediated phototactic behavior in agricultural pests and provide insights into the molecular basis of insect behavioral regulation.

## 2. Materials and Methods

### 2.1. Insects

The laboratory colony of *D. citri* (ACP) was derived from orange jasmine *Murraya paniculata* at the South China Agricultural University, Guangzhou, China, and was continuously reared on the same host plant in phytotrons. Rearing conditions were maintained at 26 ± 2 °C, 60% ± 10% relative humidity, and a photoperiod of 14 h light and 10 h dark.

### 2.2. Identification of DcTβH and DcOctβR Genes

Total RNA was extracted from the whole body of ACP adults using RNAiso Plus reagent (Takara, Beijing, China). RNA purity and concentrations were assessed by Agilent 2100 Bioanalyzer (Agilent Technologies, Palo Alto, CA, USA), NanoDrop One spectrophotometer (Thermo Fisher Scientific Inc., Waltham, MA, USA) and 1% agarose gel electrophoresis. First-strand cDNA was synthesized with the PrimeScript™ II 1st Strand cDNA Synthesis Kit (Takara, Beijing, China). The coding sequences of *DcTβH*, *DcOctβ1R* and *DcOctβ2R* were amplified using TaKaRa Ex Taq^®^ (Takara, Beijing, China), with primers designed by Primer 5.0 software (Table S1). Purified PCR products were cloned into the pMD-18-T Vector (Takara, Beijing, China) and then sequenced.

### 2.3. Sequence Alignment and Phylogenetic Analysis

Nucleic acid sequence translations of *DcTβH*, *DcOctβ1R* and *DcOctβ2R* genes were performed using DNAMAN Version9 (Lynnon Biosoft, San Ramon, CA, USA). Multiple sequence alignments of *TβH* and *Octβ1R* homologous genes were carried out using ClustalX [32] and then visualized by Jalview [33]. The deduced protein sequences were analyzed for pI and MW using the ExPASy Proteomics Server (https://web.expasy.org/compute_pi/, accessed on 14 June 2026). The signal peptide and conserved domains of *Dc*TβH were predicted and analyzed by Signal P 6.0 (https://services.healthtech.dtu.dk/services/SignalP-6.0/, accessed on 14 June 2026) and conserved domains (https://www.ncbi.nlm.nih.gov/cdd/?term=, accessed on 14 June 2026). The putative transmembrane regions of receptor *DcOctβ1R* and *DcOctβ2R* genes were predicted using TMHMM-2.0 (http://www.cbs.dtu.dk/services/TMHMM-2.0/, accessed on 14 June 2026). The deduced amino acids were visualized using IBS 2.0 [34].

To identify the potential orthologs of *DcTβH*, *DcOctβ1R* and *DcOctβ2R*, a phylogenetic analysis of these proteins was performed. Homologous sequences were obtained through a BlastP search against the non-redundant protein database on the National Center for Biotechnology Information (NCBI) website (https://www.ncbi.nlm.nih.gov/, accessed on 14 June 2026). The phylogenetic tree was constructed using MEGA 7.0 software based on the neighbor-joining method, with a bootstrap value of 1000. The sequences of genes from other insects were retrieved from the NCBI database and are listed in Table S2. The TβH of *Caenorhabditis elegans* was used as outgroup in constructing the phylogenetic analysis of DcTβH. Neither inactivation nor afterpotential E (ninaE) and phenylalanyl-methionyl-arginyl-phenylalanine amide receptor (FMRF amide receptor) of *D. melanogaster* were used as outgroups for the phylogenetic analysis of *Dc*OctβRs. The tertiary structures of DcTβH and DcOctβRs were predicted using Swiss-model (https://swissmodel.expasy.org/, accessed on 14 June 2026).

### 2.4. Expression Profiles of DcTβH and DcOctβR Genes

To investigate the relative expression levels of *DcTβH*, *DcOctβ1R* and *DcOctβ2R* genes, total RNA was isolated from various developmental stages and tissues of adults. According to the characteristics described in Ruan et al. [35], nymphs of different developmental stages and newly emerged adults of different sexes were distinguished using an optical microscope (ZEISS, Oberkochen, BW, Germany). Nymphs with different developmental stages, one-day-old virgin adults, seven-day-old virgin adults and mated adults, were collected. Virgin female and male adults were separately reared on mature leaves of *M. paniculata*. Mated adults were maintained with females and males at a 1:1 ratio. Each replicate contained 150 first and second instar nymphs, 100 third instar nymphs, 50 fourth instar nymphs and fifth instar nymphs, along with 10 adults. The heads, thorax, gut, ovary, gonad and fat body were collected from four-day-old virgin adults. For each biological replicate, 50 adults were pooled to harvest head and thorax tissues, whereas 120 adults were collected for isolation of fat body, mid gut, and gonad samples. Total RNA was isolated using RNAiso Plus reagent, and the first-strand cDNA was synthesized by PrimeScript™ RT reagent Kit with gDNA Eraser (Perfect Real Time) (Takara, Beijing, China). Specific primers for quantitative reverse transcription polymerase chain reactions (qRT-PCR) were designed with Primer 5.0 software (Table S1). The *DcActin* was used as an internal control to normalize the cDNA among the samples. The qRT-PCR reactions were carried out using TB Green^®^ Premix Ex Taq™ II (Takara, Beijing, China) and CFX 96™ Real-Time Detection System (Bio-Rad, Hercules, CA, USA), under the following conditions: initial denaturation at 95 °C for 30 s, followed by 40 cycles of 95 °C for 20 s and 60 °C for 45 s. The relative expression was calculated according to the 2^−ΔΔCt^ method [36,37]. Each treatment included three biological replicates‌.

### 2.5. Heterologous Expression of DcOctβ1R and DcOctβ2R

The *DcOctβ1R* and *DcOctβ2R* were subcloned from the pMD-18T vector (Biodragon, Suzhou, China) into the pCDNA3.1 vector using FastDigest HindIII (Thermo, Waltham, MA, USA) and the Lightening Cloning Kit. Specific primers were designed and utilized to construct *DcOctβ1R*-pCDNA3.1 and *DcOctβ2R*-pCDNA3.1 (Table S1). The plasmids of *DcOctβ1R*-pCDNA3.1 and *DcOctβ2R*-pCDNA3.1 were then extracted with TIANprep Mini Plasmid Kit (TIANGEN, Beijing, China). Subsequently, the plasmids were transfected into HEK-293T cells using Lipofectamine™ 2000 (Invitrogen, Carlsbad, CA, USA). The transfected cells were cultured in 12-well plates in an incubator (Heal Force, Shanghai, China) at 37 °C under 5% CO_2_ atmosphere. The transfection efficiency was determined by qRT-PCR. The HEK293T-*GAPDH* was used as an internal control to normalize the cDNA across samples (Table S1).

### 2.6. cAMP Determination

The transfected cells were cultured in 12-well plates for 36 h. Subsequently, they were plated at a density of 1 × 10^5^ cells per well in 96-well plates. After overnight culturing, the culture medium was removed. The ligand, agonist, and antagonist treatments were then carried out by adding 20 μL of the tested reagent and incubating at room temperature for 20 min. The intracellular cAMP levels were determined by cAMP-Glo™ Assay (Promega, Madison, WI, USA), following the manufacturer’s protocol. The ligands employed in this determination included dopamine (DA), OA, tyramine (TA), serotonin (5-HT), and histamine (HA). The agonists were clonidine, naphazoline and tolazoline, while the antagonists were yohimbine, phentolamine, epinastine, mianserin, SCH23390, spiperone, chlorpromazine and metoclopramide. To measure the stimulation of intracellular cAMP production by ligands and agonists, the cells were treated with ligands at 10 µM. Additionally, 10 µM of forskolin was used as a positive control for receptor activation. For the analysis of antagonists, the cells were treated with 10 μM antagonist in the presence of 10 nM OA. Following these treatments, the effective reagents were selected for further analysis. Each treatment included at least three biological replicates‌.

The ligands, including SCH23390, spiperone, chlorpromazine, metoclopramide and forskolin, were purchased from Shanghai Aladdin Biochemical Technology Co., Ltd., Shanghai, China. The agonists and remaining antagonists were sourced from Shanghai Yuanye Biotechnology Co., Ltd., Shanghai, China. All the tested compounds were dissolved in dimethyl sulfoxide, and further serially diluted with phosphate-buffered saline.

### 2.7. Transcript Level Analysis of DcTβH and DcOctβR Genes During Phototaxis

Seven-day-old ACP adults were prepared in this study. Specifically, adults were screened following the experimental procedures described in a previous phototaxis study [31]. The individuals exhibiting phototaxis (PH) and non-phototaxis (NP) behavioral phenotypes were collected respectively for analysis. All adult specimens were dissected on ice to isolate head tissues. The transcript levels of *DcTβH* and *DcOctβ1R* genes were quantified by qRT-PCR. Each treatment included three biological replicates. For each biological replicate, 50 randomly mixed female and male adults were pooled for head tissue collection.

### 2.8. RNA Interference

Primers containing the T7 promoter (Table S1) were used for PCR amplification, and the purified products were subsequently employed for synthesis of double-stranded RNA (dsRNA). The ds*GFP* served as the control group (Table S1). The synthesis process was carried out by using HiScribe™ T7 in vitro transcription kit (New England Biolabs Inc., Ipswich, MA, USA). The three-day-old newly emerged female and male adults of ACP were subjected to RNAi. A total of 500 ng dsRNA was injected into the ACP adults at the junction between pterothorax and wing base, with a microinjector (WPI, Sarasota, FL, USA). After being fed on *M. paniculata* for 24 h, the ACP were collected for the assessment of knockdown efficiency and behavioral assays. The knockdown efficiency was determined by qRT-PCR, which followed the same methodology as that used for determining expression profiles of *DcTβH*, *DcOctβ1R* and *DcOctβ2R* genes. Each treatment included three biological replicates, with ten individuals tested per replicate.

### 2.9. Phototactic Behavioral Assay

The phototactic behavioral assay was conducted in a black chamber (90 cm × 60 cm × 60 cm) to eliminate external light interference. A Y-maze apparatus (arm length: 7.00 cm; inner diameter: 1.80 cm; outer diameter: 2.20 cm) was placed inside the chamber, consisting of three arms: a basic arm, a positive phototactic arm, and a negative phototactic arm. A light-emitting diode light with a wavelength of 400 nm was mounted above the chamber. The light intensity was measured using a portable irradiatometer (FZ-A, Beijing Shida Photoelectric Technology Co., Ltd., Beijing, China), and the height between the light source and the Y-tube was maintained at a fixed distance. All adults underwent 1 h of dark adaptation before testing. Ten adults were introduced to the terminal end of the basic arm, and the 30 min behavioral assay was initiated. An adult was defined to make a valid directional choice if its entire body entered the terminal section of either selective arm (positive or negative phototactic arm) at any time within the 30 min trial. Individuals that stayed within the basic arm throughout were recorded as no-response individuals. The number of adults in the phototactic arm was recorded at 2, 5, 10, 15, 20, 25, and 30 min during assay. Each experiment included ten biological replicates.

### 2.10. Statistical Analysis

Statistical analysis and graphical visualization were carried out using SPSS 25.0 (IBM, Armonk, NY, USA) and GraphPad Prism version 8.0.2 (GraphPad Software, San Diego, CA, USA). The relative transcript levels were quantified and subjected to statistical analysis via one-way ANOVA, followed by Tukey’s post hoc test. In RNAi experiments, differences in relative transcript levels were evaluated using Student’s *t* test. Pharmacological comparisons were assessed by one-way ANOVA with Dunnett’s multiple comparison test. The positive phototactic ratio (PR) was computed as the number of adults in the positive phototactic arm divided by the total number of individuals with valid directional choices. PR data were transformed using the formula y = arcsin(sqrt(PR)) to meet the assumptions of normality and homogeneity of variances. The transformed data (y) were then analyzed for statistical significance through repeated-measures ANOVA followed by Tukey’s post hoc test. The EC_50_ and IC_50_ values were ascertained by nonlinear regression analysis using the normalized response-variable slope model. Statistical significance was set at *p* < 0.05.

## 3. Results

### 3.1. Molecular Cloning and Sequence Analysis of DcTβH, DcOctβ1R and DcOctβ2R

**Figure 1:**
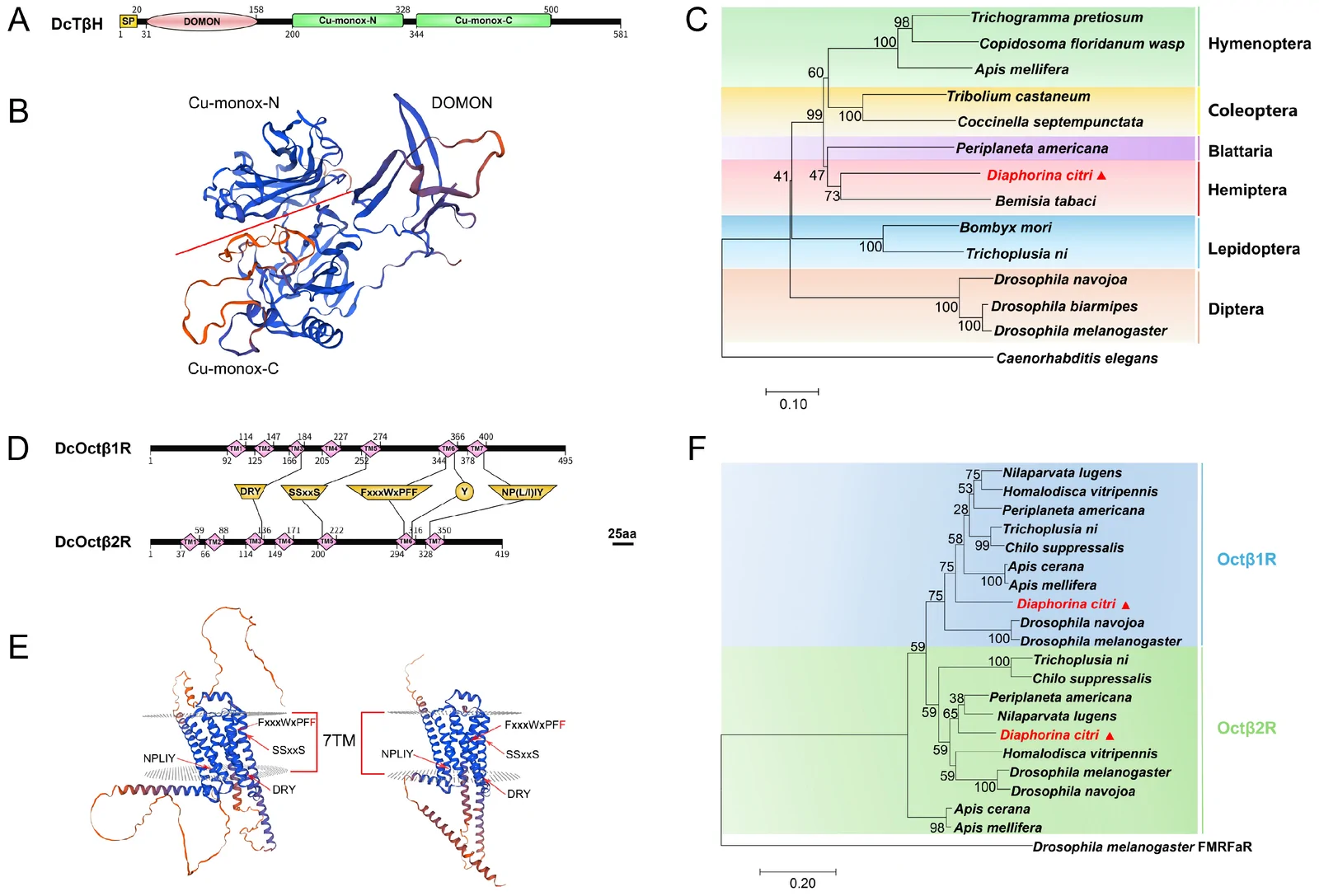
Structural characterization and phylogenetic analysis of DcTβH, DcOctβ1R and DcOctβ2R. (**A**,**D**) Conserved domain architectures and functionally critical amino acid residues of DcTβH (**A**), DcOctβ1R and DcOctβ2R (**D**). Residues marked with yellow trapezoids and circles represent conserved functional sites essential for OctβRs activity. SP, signal peptide; DOMON, Domon-like ligand-binding domain; Cu-monox-N and Cu-monox-C, N-terminal domain and C-terminal domain of copper type II ascorbate-dependent monooxygenase; TM, transmembrane domain. (**B**,**E**) Predicted tertiary structures of DcTβH (**B**), DcOctβ1R and DcOctβ2R (**E**). Confidence of structural prediction is indicated by a color gradient ranging from high confidence (blue) to low confidence (red). (**C**,**F**) Phylogenetic analysis of TβH proteins (**C**) and two *Dc*OctβRs (**F**) from *Diaphorina citri* and other insect species.

The cDNAs encoding *DcTβH*, *DcOctβ1R* and *DcOctβ2R* were cloned. The coding sequence of *DcTβH* was 1746 bp, encoding 581 amino acids with a calculated molecular weight (MW) of 66.4 kDa and a theoretical predicted isoelectric point (pI) of 5.49. Its deduced amino acid sequence had a signal peptide (probability = 0.974817), indicating a secreted protein (Figure 1A). In addition, it contained three conserved domains: DOMON, copper type II ascorbate-dependent monooxygenase N-terminal (Cu-monox-N), and C-terminal (Cu-monox-C) (Figure 1A,B). The sequence alignment showed *Dc*TβH shared 35.63–54.14% similarity with TβH of *Periplaneta americana*, *Trichogramma pretiosum* and *Bemisia tabaci*, *D. melanogaster* and *Apis mellifera* (Figure S1; Table S3). Furthermore, phylogenetic analysis revealed that *Dc*TβH clustered with TβH of *B. tabaci* (Figure 1C).

The coding sequences of *DcOctβ1R* and *DcOctβ2R* were 1488 bp and 1260 bp, encoding 495 and 419 amino acids. Their predicted MWs were 55.9 kDa and 48.1 kDa, with pIs of 9.17 and 8.81, respectively. Both DcOctβ1R and DcOctβ2R had typical GPCR features, including seven transmembrane (TM) domains and conserved motifs (Figure 1D,E). According to the nomenclature of the Ballesteros–Weinstein system [38], conserved residues like DRY in TM3, serine residue (S) in the SS××S motif region of TM5, and Tyr residue (Y) in TM6 are crucial for ligand binding and G protein coupling [39]. Two phenylalanine residues (F) followed the F×××W×P motif in TM6, with the second F unique to aminergic GPCRs [40,41]. The NP(L/I)IY motif in TM7 is also conserved in rhodopsin GPCRs [42]. The sequence alignment showed DcOctβ1R shared 47.51–60.90% similarity with Octβ1R of *Nilaparvata lugens*, *Tribolium castaneum*, *P. americana*, *D. melanogaster*, *A. mellifera*, while DcOctβ2R shared 62.17–74.83% similarity with Octβ2R of these species (Figure S1; Table S3). The phylogenetic analysis of DcOctβ1R and DcOctβ2R showed that DcOctβ1R and DcOctβ2R were closely related to their orthologous receptors, respectively (Figure 1F). DcOctβ1R exhibited a closer phylogenetic relationship with *Drosophila* Octβ1R homologs rather than with hemipteran homologs from *N. lugens* and *Homalodisca vitripennis*. DcOctβ2R showed a close relationship with *N. lugens* and *P. americana* (Figure 1F).

### 3.2. Spatial and Temporal Expression Profiles of DcTβH, DcOctβ1R and DcOctβ2R

**Figure 2:**
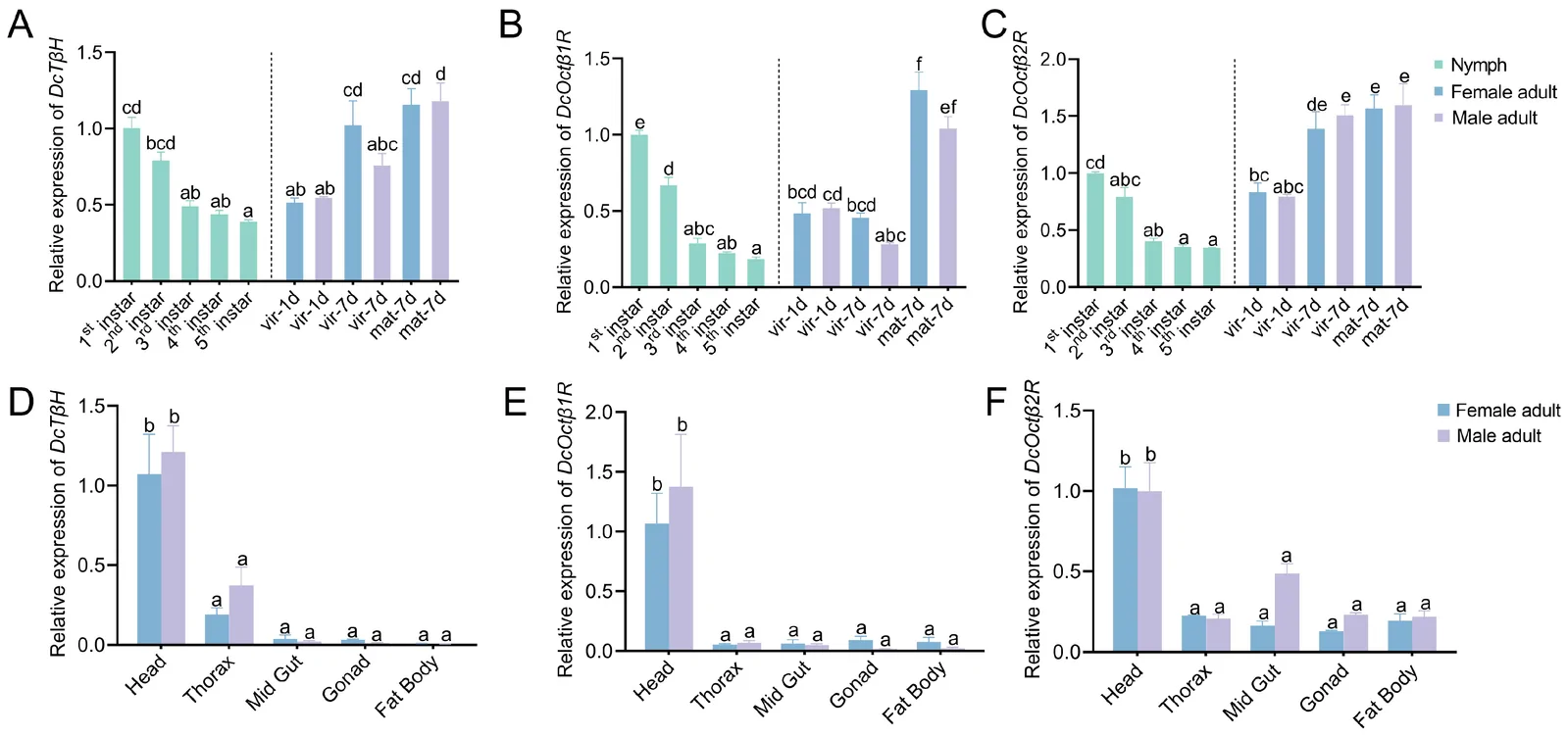
Relative expression levels of *DcTβH*, *DcOctβ1R* and *DcOctβ2R* at different developmental stages and in various tissues. (**A**–**C**) Relative expression levels of *DcTβH*, *DcOctβ1R* and *DcOctβ2R* in nymphs from the first to the fifth instar and in adults at different developmental phases. The one-day-old/seven-day-old virgin (vir-1d/7d) female and male adults were reared separately. The mated adults were paired at 1:1 sex ratio and fed for seven days (mat-7d). (**D**–**F**) The relative expression levels of *DcTβH*, *DcOctβ1R* and *DcOctβ2R* in different tissues of four-day-old virgin female and male adults. Different letters above the bars indicate significant differences at *p* < 0.05 (Tukey’s post hoc test). Data are the means of three replicates ± SEM.

The stage- and tissue-specific expression analysis of *DcTβH*, *DcOctβ1R*, and *DcOctβ2R* genes showed that the expression levels of these genes declined during nymphal development and rose after emergence to adulthood during maturation (*DcTβH*: *F*_10,22_ = 14.171, *df* = 10, *p* < 0.001; *DcOctβ1R*: *F*_10,22_ = 47.926, *df* = 10, *p* < 0.001; *DcOctβ2R*: *F*_10,22_ = 27.998, *df* = 10, *p* < 0.001). They showed higher expression levels in the first and second instar nymphs compared to the third to fifth instar nymphs (Figure 2A–C). Among them, only *DcOctβ1R* had significantly higher expression levels in the first instar nymphs than the second instar (Figure 2B). After adult emergence, *DcTβH* had comparable transcript levels in one-day-old adults and the second to fifth instar nymphs (Figure 2A). With adult development to the seventh day, the expression level of *DcTβH* increased, except for virgin male adults (Figure 2A). For *DcOctβ1R*, its transcript levels were comparable in virgin adults and the third to fifth instar nymphs (Figure 2B). Additionally, in 7-day-old adults, mated females and males exhibited 2.83-fold- and 3.71-fold-higher expression relative to virgin counterparts, respectively (Figure 2B). For *DcOctβ2R*, expression levels were significantly higher in 7-day-old adults than in 1-day-old adults, and mating status exerted no significant influence on its transcription (Figure 2C).

In adult tissues, the highest expression levels of all three genes were consistently detected in the head, and there were no significant sex differences (*DcTβH*: *F*_9,20_ = 20.229, *df* = 9, *p* < 0.001; *DcOctβ1R*: *F*_9,20_ = 9.406, *df* = 9, *p* < 0.001; *DcOctβ2R*: *F*_9,20_ = 20.266, *df* = 9, *p* < 0.001). (Figure 2D–F). The transcript levels of *DcTβH* in the head were 3.24–5.63-fold higher than those in the thorax; relative to the mid gut, gonad and fat body, head *DcTβH* expression was markedly elevated by 27.70–208.90-fold (Figure 2D). For *DcOctβ1R*, expression was low in the thorax, mid gut, gonad and fat body, with head expression 11.86–78.08-fold higher (Figure 2E). Unlike *DcTβH* and *DcOctβ1R*, *DcOctβ2R* exhibited milder expression differences between the head and other tissues (Figure 2F). The largest expression difference was found between the head and female gonad, with a 7.87-fold change (Figure 2F).

### 3.3. Heterologous Expression and Functional Assay of DcOctβ1R and DcOctβ2R

**Figure 3:**
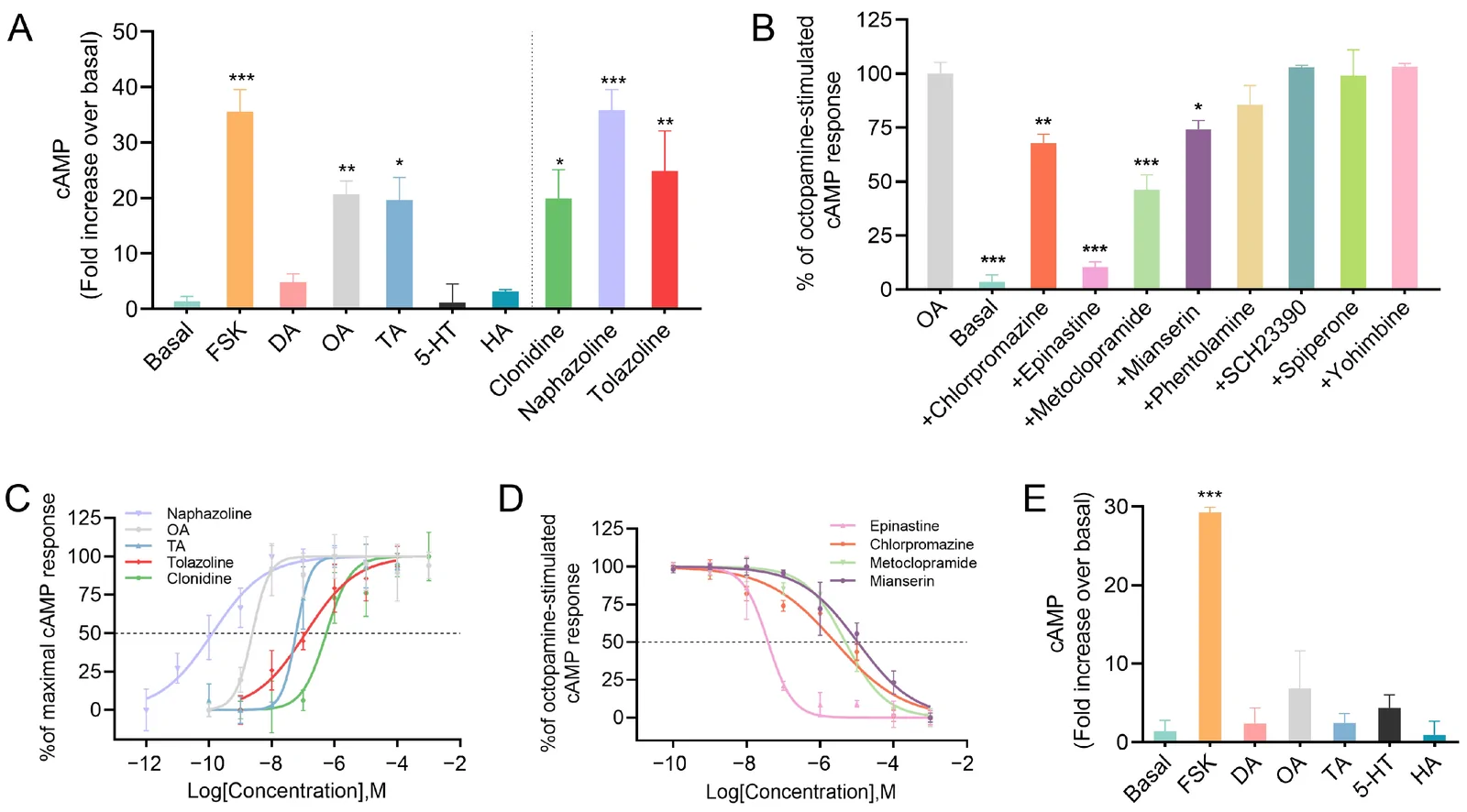
Effects of various agonists and antagonists on intracellular cAMP levels in HEK-293T cells transiently expressing DcOctβ1R and DcOctβ2R. (**A**) Intracellular cAMP accumulation in *Dc*Octβ1R-expressing cells subjected to five ligands and three agonists at a concentration of 10 μM. The basal level refers to the intracellular cAMP level in cells without application of any ligand or agonist. (**B**) Effects of antagonists at a concentration of 10 μM on OA-stimulated (10 nM) intracellular cAMP production in DcOctβ1R-expressing cells. The basal level represents the intracellular cAMP level of cells without application of any ligand or antagonist. The amount of cAMP is given as a percentage of the value achieved with 10 nM OA (100%). (**C**) Dose–response curves of OA/TA and effective agonists in DcOctβ1R-expressing cells. The values are calculated to the maximal cAMP response (=100%) for each agonist. (**D**) Dose–response curves of effective antagonists in DcOctβ1R-expressing cells. The amount of cAMP is given as a percentage of the value achieved with 10 nM OA (100%). (**E**) Intracellular cAMP accumulation in DcOctβ2R-expressing cells subjected to five ligands at a concentration of 10 μM. The basal level refers to the intracellular cAMP level in cells without application of any ligand or agonist. Statistical significance was determined using one-way ANOVA followed by Dunnett’s multiple comparison test. Asterisks indicate values significantly different from control conditions; in (**A**) and (**E**) control is basal cAMP level, and in (**B**) control is OA-stimulated cAMP level (* *p* < 0.05, ** *p* < 0.01, *** *p* < 0.001). Data are presented as means ± SEM of at least three experimental replicates.

Transient expression of *Dc*Octβ1R and *Dc*Octβ2R was achieved in HEK-293T cell lines, as confirmed by qRT-PCR analysis of mRNA levels (Figure S2). OA, TA and all agonists (clonidine, naphazoline, and tolazoline) at a concentration of 10 µM evaluated in this assay could significantly induce cAMP accumulation in cells expressing DcOctβ1R, as well as forskolin (FSK) treatment compared with the control condition (*F*_9,30_ = 12.428, *df* = 9, *p* < 0.001; Figure 3A). DA, 5-HT and HA failed to induce intracellular cAMP elevation (Figure 3A). To test whether candidate antagonists block OA-triggered signaling, cells were pre-incubated with each antagonist at 10 µM prior to stimulation with 10 nM OA. The results showed that epinastine, mianserin, metoclopramide and chlorpromazine could attenuate the OA-induced cAMP elevation (*F*_9,22_ = 39.892, *df* = 9, *p* < 0.001; Figure 3B). In contrast, phentolamine, SCH23390, spiperone and yohimbine had no significant effects on OA-induced cAMP levels (Figure 3B).

Further, the increasing concentrations of OA, TA, three potent agonists and four antagonists were applied to the cells, and dose–response curves for DcOctβ1R are displayed (Figure 3C,D). Among these biogenic amines and their potent agonists, naphazoline exhibited the highest potency (EC_50_ = 1.29 × 10^−10^ M), followed by OA (EC_50_ = 2.40 × 10^−9^ M), TA (EC_50_ = 5.93 × 10^−8^ M), tolazoline (EC_50_ = 1.20 × 10^−7^ M), and clonidine (EC_50_ = 5.45 × 10^−7^ M) in descending order (Figure 3C, Table S4). Among the examined antagonists, epinastine displayed the strongest inhibitory efficacy. The potency ranking was as follows: epinastine (IC_50_ = 3.80 × 10^−8^ M) > chlorpromazine (IC_50_ = 2.56 × 10^−6^ M) > metoclopramide (IC_50_ = 4.90 × 10^−6^ M) > mianserin (IC_50_ = 1.06 × 10^−5^ M) (Figure 3D, Table S5). Notably, the results demonstrated that cells expressing *Dc*Octβ2R remained stably unresponsive to cAMP induction, regardless of treatment with DA, OA, TA, 5-HT or HA (Figure 3E).

### 3.4. DcTβH and DcOctβ1R Are Involved in Regulating Phototactic Behavior in ACP

**Figure 4:**
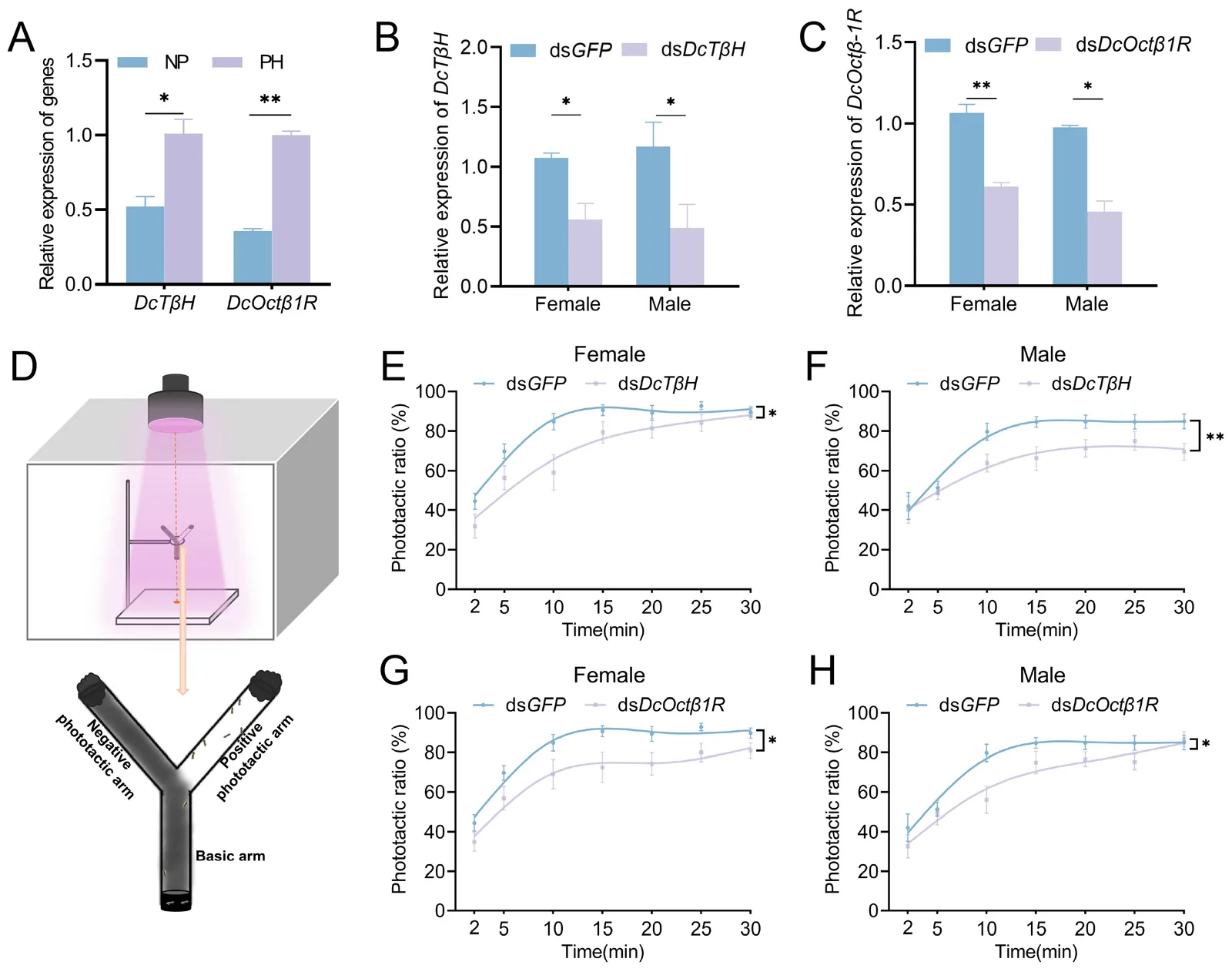
Expression profiles and functional characterization of *DcTβH* and *DcOctβ1R* in phototactic behavior of ACP. (**A**) Transcript levels of *DcTβH* and *DcOctβ1R* in heads of adults with phototaxis (PH) and non-phototaxis (NP). (**B**,**C**) Relative expression of *DcTβH* (**B**) and *DcOctβ1R* (**C**) after dsRNA treatment for 24h. The statistical difference between the treatment and control group is indicated by the asterisks (* *p* < 0.05, ** *p* < 0.01, Student’s *t* test). Data are presented as means of three replicates ± SEM. (**D**) The diagram of phototactic behavioral assay. The diagram on the right represents distribution of *D. citri* individuals in the Y-maze apparatus while the phototactic behavior occurs. The light intensity was measured at the orange spot, which was 300 μW/cm^2^. (**E**–**H**) The phototactic ratio of adults after dsRNA treatments. The statistical difference between the treatment and control group is indicated by the asterisks (* *p* < 0.05, ** *p* < 0.01, repeated-measures ANOVA followed by Tukey’s post hoc test). Data are presented as means of ten replicates ± SEM.

To investigate the involvement of OA in the phototactic behavior of ACP, the expressions of *DcTβH* and *DcOctβ1R* genes were determined during the phototactic behavior. The results showed that the *DcTβH* (*t* = 4.175, *df* = 4, *p* = 0.014) and *DcOctβ1R* (*t* = 20.795, *df* = 4, *p* < 0.001) genes were significantly upregulated in the heads of phototactic adults relative to non-phototactic individuals (Figure 4A). When the two genes were knocked down by RNAi in 3-day-old ACP adults, the *DcTβH* expression decreased 1.92-fold in ds*DcTβH*-treated females (*t* = 3.630, *df* = 4, *p* = 0.022) and 2.76-fold in males (*t* = 3.017, *df* = 4, *p* = 0.039) compared to ds*GFP* controls (Figure 4B). Meanwhile, the *DcOctβ1R* expression was reduced by 1.74-fold in ds*DcOctβ1R*-treated females (*t* = 8.100, *df* = 4, *p* = 0.001) and 1.96-fold in males (*t* = 4.466, *df* = 4, *p* = 0.011) (Figure 4C). Notably, effective knockdown of *DcOctβ2R* was not achieved via dsRNA treatment, even though two independent dsRNA fragments targeting different regions of the gene were designed and tested (Figure S3). Subsequently, the phototactic behavior assay was conducted for 30 min (Figure 4D). ACP injected with ds*DcTβH* or ds*DcOctβ1R* exhibited lower PR values than the ds*GFP* control group (Figure 4E–H). RNAi treatment significantly dampened phototactic behavior in both sexes compared with ds*GFP* treatment. Nevertheless, the overall temporal trend of phototactic responses did not differ among treatment groups (ds*DcTβH*: female: *F*_6,108_ = 1.319, *p* = 0.255; male: *F*_6,108_ = 1.575, *p* = 0.161; ds*DcOctβ1R*: female: *F*_6,108_ = 0.626, *p* = 0.662; male: *F*_6,108_ = 1.647, *p* = 0.084). All groups followed similar temporal dynamics: PR increased rapidly within the initial 15 min and then plateaued. Females injected with ds*DcTβH* responded rapidly, with PR reduced significantly by 12.6% at 2 min and reaching the maximum reduction of 25.8% at 10 min; the intergroup difference diminished as time elapsed (*F*_1,18_ = 5.619, *df* = 1, *p* = 0.029; Figure 4E). By contrast, males injected with ds*DcTβH* showed delayed but stable inhibition (*F*_1,18_ = 8.633, *df* = 1, *p* = 0.009; Figure 4F). The reduction peaked at 18.6% at 15 min and remained 15.6% at 30 min. For ds*DcOctβ1R*-injected ACP, females displayed gradual and stable inhibition, with a reduction ranging from 8.9% to 18.2% throughout the entire 30 min (*F*_1,18_ = 4.666, *df* = 1, *p* = 0.044; Figure 4G). Males had gradual inhibition followed by partial recovery (*F*_1,18_ = 5.680, *df* = 1, *p* = 0.028; Figure 4H). The reduction reached its peak of 23.7% at 10 min and declined gradually afterwards.

## 4. Discussion

Given the complexity of behavioral regulation, investigating the central nervous system may provide crucial insights. Among the key neuromodulators, biogenic amines and their physiological roles in insects have been widely studied [43,44,45,46]. OA is known to influence diverse functions, including reproduction [18,20], aggressive behavior [20], and locomotion [22]. To elucidate its role, research has focused on OA synthesis and its receptors, particularly OctβRs, which have been identified in multiple species such as *D. melanogaster* [47], *Apis mellifera* [48], *Tribolium castaneum* [22]. While some studies have explored the molecular characterization of OA receptors to identify new pest control targets [49,50,51,52], research on TβH remains limited, having been mainly characterized in model insects [53,54,55,56,57] and in *Mythimna separata* [58]. In this study, *DcTβH*, *DcOctβ1R* and *DcOctβ2R* were identified and subjected to functional characterization. Our results suggest that the OA positively regulates phototactic behavior via *DcOctβ1R* in ACP, thereby enhancing our understanding of OA-mediated phototactic behavior in agricultural pests.

The expression profiles of *DcTβH, DcOctβ1R*, and *DcOctβ2R* genes displayed a U-shaped pattern across the developmental stages of ACP, consistent with findings in other species such as *Bactrocera dorsalis* [51], *Laodelphax striatellus* [28], and *Plutella xylostella* [59]. This conservation supports the involvement of OA signaling in the insect developmental progression. Notably, this expression profile emphasizes the importance of these genes in the early nymphal stages and mature adult stages. These are critical phases for ACP dispersal and host colonization, during which phototaxis also plays a role. Notably, *DcOctβ1R* expression was higher in mated adults than in virgins, which is consistent with the established role of OA in regulating insect reproduction [18,60,61]. This highlights its potential role in ACP reproduction, as OA is known to modulate insect reproductive processes [22,27,62,63]. Since phototaxis is an important cue for insect reproduction [2], exploring shared molecular regulatory nodes that coordinate both phototactic and reproductive behaviors could be pursued. Furthermore, the high expression of these genes in the head is in line with insect biology and the findings in other species [22,28,61], suggesting their potential functional significance in the central nervous system and the neuromodulation of insect behaviors.

Pharmacological characterization of DcOctβ1R revealed the following rank order of agonist potency: naphazoline > OA > TA > tolazoline > clonidine. The potency sequence from naphazoline to TA aligns with findings in BdOctβR1 [51] and CcOctβR1 [64]. However, an intriguing distinction emerged with clonidine, which demonstrated higher efficacy than tolazoline in CcOctβR1 [64]. Regarding antagonist potency, the following hierarchy was established, epinastine > chlorpromazine > metoclopramide > mianserin, which differs slightly from the sequence reported for CcOctβR1 (epinastine > mianserin > metoclopramide > chlorpromazine) [64]. These pharmacological profiles provide molecular information to aid the development of novel insecticidal target agents and may serve as preliminary reference data for future investigations on combined chemical and physical pest control regimes.

In contrast, regardless of ligand treatment, cells expressing DcOctβ2R showed no detectable cAMP response upon stimulation with either OA or TA. Two mechanistic hypotheses may account for this finding. First, mutations or sequence variations in the receptor coding region could induce structural alterations, resulting in protein misfolding or impaired plasma membrane localization of the receptor protein [65]. Second, biogenic amine receptors exert functions by triggering intracellular signaling cascades that modulate cAMP and/or intracellular calcium. Closely related aminergic GPCRs have been reported to exhibit G protein subtype switching, whereby signaling no longer proceeds through the cAMP-dependent pathway [66]. This mechanism aligns with the lack of detectable cAMP changes observed in our assay system. Furthermore, two dsRNA fragments targeting distinct regions of *DcOctβ2R* failed to achieve effective transcript knockdown in ACP. In general, RNAi efficiency is susceptible to multiple experimental variables, including dsRNA dosage and concentration, insect developmental stages, target tissue characteristics, the structural type and length of delivered dsRNA fragments, exposure duration, and sampling time points [67]. Therefore, systematic optimization of RNAi experimental conditions is still required to achieve effective knockdown and further clarify the physiological function of *DcOctβ2R* in ACP. We hypothesize that this silencing inefficiency may be attributed to the presence of multiple endogenous alternatively spliced transcript variants of this gene. If the two selected dsRNA target sequences only cover a small subset of these isoforms, the overall RNAi silencing effect would be substantially weakened. Notably, this explanation remains speculative and requires further experimental validation in future studies. Collectively, the functional role of *DcOctβ2R* in regulating ACP physiological behaviors remains unclear and warrants further in-depth investigation in future studies.

Insect phototactic behavior is an integrated sensorimotor behavior that couples visual perception with locomotor output. Studies have demonstrated that high levels of serotonin inhibit phototactic behavior in *D. melanogaster* [68,69] and *Apis mellifera* [70]. This phototactic behavior, which is affected by circadian photosensitivity, diversifies phototactic polarity. Similarly, it is hypothesized that high levels of OA inhibit phototactic behavior in *Apis mellifera* based on individual differences in sensory responsiveness [30]. However, DA-deficient flies in *D. melanogaster* are also defective in positive phototaxis [69]. These findings indicate that the regulation of phototaxis by different biogenic amines varies among species and follows different regulatory pathways. In this study, the temporal dynamics of the phototactic ratio over 30 min were characterized in ACP. The phototactic ratio of control individuals reached a plateau at 30 min, while knockdown of *DcTβH* or *DcOctβ1R* mainly reduced the phototactic ratio during the rapid ascending phase (10–15 min). This suggests that OA plays a positive regulatory role in the phototactic behavior of ACP. Notably, inconsistent effects of OA on phototaxis have been reported in honeybees: injection of OA into the median ocellus enhances phototaxis, whereas dietary OA administration exerts an inhibitory effect [29,30]. These discrepancies are probably attributable to differences in delivery routes and experimental paradigms. The results obtained from feeding experiments may also be confounded by the roles of OA in colony division of labor and foraging behavior. Previous studies verified the findings through electroretinography [29,30], providing more direct physiological evidence. In contrast, their results might have been affected by colony division of labor and foraging behavior, since OA is known to regulate these processes [71,72]. In comparison, our current study is limited to behavioral phenotyping and lacks direct electrophysiological evidence. Considering the highly complex neural regulatory network underlying insect phototaxis, which covers photoreceptor spectral sensitivity, neural signal integration, and context-dependent behavioral decision-making, the exact mechanistic pathway by which OA modulates ACP phototactic behavior remains unclear and speculative. Future studies integrating electroretinography and behavioral trajectory analysis will help uncover the mechanistic basis of OA-mediated phototactic regulation in ACP and provide more direct and rigorous physiological evidence. Meanwhile, all phototaxis assays in this study relied exclusively on 400 nm light; whether OA mediates ACP phototactic responses to other wavelengths remains unclarified. Further behavioral tests covering other wavelengths are warranted to fully dissect the core mechanisms underlying OA-dependent phototactic plasticity and the development of multi-wavelength light-trapping strategies for ACP. In addition to the above limitations, the present study advances the mechanistic understanding of OA signaling modulation of phototactic behavior in ACP and provides a mechanistic foundation for the future optimization of light-trapping and pest management strategies. As a vital neurotransmitter, OA participates in the regulation of multiple insect behaviors, with reproductive activity serving as one plausible downstream behavioral output. Considering the close biological association between phototactic behavior and reproductive behavior [3], OA-related molecular indicators may be developed into convenient biomarkers to monitor the mating status of field psyllid populations trapped by light traps.

## 5. Conclusions

This study identified and characterized three core genes in the OA signaling pathway in *D. citri*: *DcTβH*, *DcOctβ1R*, and *DcOctβ2R*. All three genes exhibited a U-shaped developmental expression pattern and high transcript abundance in the head, suggesting potential involvement in the early nymphal and adult stages and essential roles in the central nervous system. Pharmacological assays revealed that OA and TA induced cAMP accumulation via DcOctβ1R, which also exhibited differential potency against the tested agonists and antagonists. Behavioral assays demonstrated that silencing *DcTβH* or *DcOctβ1R* significantly impaired the ACP phototactic behavior. These findings revealed that OA signaling positively regulates phototactic behavior via *DcOctβ1R* in the ACP. This study advances the understanding of OA signaling-mediated phototactic behavior in agricultural pests and provides a foundation for exploring the molecular mechanisms of insect behaviors.

## Data Availability

The sequence data of *DcTβH*, *DcOctβ1R* and *DcOctβ2R* identified in this study have been submitted to the GenBank database under accession numbers (PV091843, PV091844, PV091845).

## Supplementary Materials

The following supporting information can be downloaded at https://www.mdpi.com/article/10.3390/insects17080851/s1, Figure S1: Amino acid sequence alignment of *DcTβH* (A), *DcOctβ1R* (B) and *DcOctβ2R* (C) with orthologous genes; Figure S2: The relative expression of *DcOctβ1R* (A) and *DcOctβ2R* (B) in HEK-293T cells; Figure S3: Relative expression of *DcOctβ2R* after ds*DcOctβ2R* treatment for 24 h; Table S1: The nucleotide sequences of primers used in this study; Table S2: The accession numbers of the sequences used in this study; Table S3: The similarity of other species shares with *Diaphorina citri* in multiple alignment; Table S4: EC_50_ and LogEC_50_ values for stimulation of cAMP response by various agonists in HEK-293T cells expressing *DcOctβ1R*; Table S5: IC_50_ and LogIC_50_ values for inhibition of OA-induced cAMP response by various antagonists in HEK-293T cells expressing *DcOctβ1R*.
