# Supplementary material for: Octopamine Signaling Regulates Phototactic Behavior via a β-Adrenergic-like Receptor in Diaphorina citri
Source: Insects. 2026 Aug 15;17(8):851. doi: 10.3390/insects17080851 (PMC13513256; doi:10.3390/insects17080851)
Supplement: Supplementary file 1 [file insects-17-00851-s001.zip › insects-4436435-supplementary.pdf]

Supplementary material

Octopamine Signaling Regulates Phototactic Behavior via a β-Adrenergic-like Receptor in *Diaphorina citri*

Fei-Feng Wang,[1,2] Xiu-Qi Zou,[2] Ming Zhong,[2] Sheng-Feng Tu,[2] Min-Er Li,2 Wen-Feng Zhao,[2] Bao-Li Qiu,[1,]* Wen Sang [2,]*

[1] Engineering Research Center of Biotechnology for Active Substances, Ministry of Education, Chongqing Normal University, 401331 Chongqing, China

[2] South China Agricultural University/Engineering Research Center of Biological Control, Ministry of Education, 510640 Guangzhou, China

[*] Correspondence: sangwen@scau.edu.cn (W. Sang); baoliqiu@cqnu.edu.cn (B.-L. Qiu)

Table S1. The nucleotide sequences of primers used in this study.

Gene	Forward primer 5’–3’	Reverse primer 5’– 3’	Product length (bp)	Position
**Primers used for open reading frame amplification**				
DcTβH	AATGTTTCTTAGGGAACTTGGC	CAGGTGGTTGTAGTCGTGTTGT	-	-
DcOctβ1R	CTCAGTAGAACCCCTCCCT	TCAAAACGTGGGTTCCA	-	-
DcOctβ2R	ATGACGGACTCCAGCAACCTCTTCG	TCATAAGCTCGACCCG	-	-
**Primers used for qRT-PCR**				
DcTβH	GAATCGGGACAACCAT	GTCGCTACTCACTGAACTCT	-	-
DcOctβ1R	CTACTCACCGAAGCCAAC	CGCAAGACTCACGACAA	-	-
DcOctβ2R	GAGGACGCCCATCACAGT	CAGGAAGAAGGGAAGCCAG	-	-
DcActin	CCCTGGACTTTGAACAGGAA	CTCGTGGATACCGCAAGATT	-	-
HEK293T-GADPH	GGTGAAGGTCGGTGTGAACG	CTCGCTCCTGGAAGATGGTG	-	-
**Primers used for vector construction, to heterologous expression of octopamine receptor**				
DcOctβ1R	[a]GGATGACGACGATAAGACTACAATGAGTGTTATGCC	GATCCGAGCTCGGTACCATCAAAACGTGGGTT	-	-
DcOctβ2R	GACGACGATAAGACTACAATGACGGACTCCAGCAACCT	TCCGAGCTCGGTACCATCATAAGCTCGACCCGAATTC	-	-
**Primers used for dsRNA synthesis**				

dsDcTβH	[b]*TAATACGACTCACTATAGGG*GTCAGCCATACAACCG	*TAATACGACTCACTATAGGG*TGGGACAGCCATCTT	388	699-1086
dsDcOctβ1R	*TAATACGACTCACTATAGGG*CAAGTCCCGCCTGTT	*TAATACGACTCACTATAGGG*GTGCTGTCGCTGATTG	393	603-995
dsDcOctβ2R 1st	*TAATACGACTCACTATAGGG*TCATCTGTGTTGCATCAGTGTG	*TAATACGACTCACTATAGGG*TGCTTCAGGTAGGTGTCCG	849	366-1214
dsDcOctβ2R 2nd	*TAATACGACTCACTATAGGG*TGCTTCTCTTGACGGTTGG	*TAATACGACTCACTATAGGG*CTTCGTGGTTTCGTTGGC	483	210-692
dsGFP	*TAATACGACTCACTATAGGG*CTTGTACAGCTCGTCCATGC	*TAATACGACTCACTATAGGG*GACGACGGCAACTACAAGAC	411	989-1399

[a]The underlined nucleotide sequence is 16 bp overlaps with the pCDNA3.1-FLAG-N vector.

[b]The italic nucleotide sequence is the T7 promoter sequence.

Table S2. The accession numbers of the sequences used in this study.

Gene	Species	Accession Number
Tyramine beta-hydroxylase	*Diaphorina citri*	PV091843
	Trichogramma pretiosum	XP_014231771.1
	Bemisia tabaci	XP_018907091.1
	Copidosoma floridanum	XP_014216871.1
	Periplaneta americana	AFO63080.1
	Apis mellifera	DAA05780.1
	Drosophila navojoa	XP_030245696.1
	Drosophila biarmipes	XP_043949511.1
	Drosophila melanogaster	Q86B61.1
	Bombyx mori	BAK09201.1
	Tribolium castaneum	XP_974169.1
	Trichoplusia ni	XP_026739132.1
	Caenorhabditis elegans	NP_001257265.1
	Coccinella septempunctata	XP_044766667.1
Octopamine receptor beta-1R	*Diaphorina citri*	PV091844
	Periplaneta americana	XP_069694870.1
	Nilaparvata lugens	ATY68966.1
	Homalodisca vitripennis	XP_046667431.1
	Helicoverpa armigera	XP_063896322.1
	Trichoplusia ni	XP_026729368.1
	Chilo suppressalis	AGV79326.1
	Dalotia coriaria	XP_065155721.1
	Cryptotermes secundus	XP_033609947.1
	Tribolium castaneum	NP_001280514.1
	Apis cerana	XP_016921836.1
	Apis mellifera	XP_397139.3
	Drosophila navojoa	XP_030242457.1

	Drosophila melanogaster	Q9VCZ3.1
Octopamine receptor beta-2R	*Diaphorina citri*	PV091845
	Periplaneta americana	XP_069695476.1
	Nilaparvata lugens	XP_039280502.1
	Nephotettix cincticeps	QID89710.1
	Drosophila melanogaster	Q4LBB9.2
	Drosophila navojoa	XP_017969142.1
	Homalodisca vitripennis	KAG8316974.1
	Helicoverpa armigera	XP_063896258.1
	Trichoplusia ni	XP_026729763.1
	Chilo suppressalis	AEO89318.1
	Dalotia coriaria	XP_065155743.1
	Tribolium castaneum	NP_001280501.1
	Cryptotermes secundus	XP_023721065.1
	Apis cerana	XP_016908111.1
	Apis mellifera	XP_006558130.1
FMRFamide receptor	*Drosophila melanogaster*	NP_647758.1
neither inactivation nor after potential E	*Drosophila melanogaster*	AAF55712.1

Table S3. The similarity of other species shares with *Diaphorina citri* in multiple alignment.

Gene	Species	Accession Number	Identity (%)
Tyramine beta-hydroxylase	*Bemisia tabaci*	XP_018907091.1	53.18
	Tribolium castaneum	XP_974169.1	54.14
	Periplaneta americana	AFO63080.1	53.70
	Drosophila melanogaster	Q86B61.1	44.75
	Apis mellifera	DAA05780.1	35.63
Octopamine receptor beta-1R	*Nilaparvata lugens*	ATY68966.1	58.56
	Tribolium castaneum	NP_001280514.1	53.88
	Periplaneta americana	XP_069694870.1	60.9

	Drosophila melanogaster	Q9VCZ3.1	47.51
	Apis mellifera	XP_397139.3	58.18
Octopamine receptor beta-2R	*Nilaparvata lugens*	XP_039280502.1	72.64
	Tribolium castaneum	NP_001280501.1	67.79
	Periplaneta americana	XP_069695476.1	74.83
	Drosophila melanogaster	Q4LBB9.2	62.17
	Apis mellifera	XP_006558130.1	68.54

Table S4. EC_{50} and $LogEC_{50}$ values for stimulation of cAMP response by various agonists in HEK 293T cells expressing DcOctβ1R.

Agonists	EC_{50} (M)	95% CI for EC_{50} (M)	Log EC_{50} (mean ± SEM)	Fitting R^2
Octopamine	2.40×10^{-9}	[1.25×10^{-9}, 4.60×10^{-9}]	-8.62 ± 0.116	0.971
Tyramine	5.93×10^{-8}	[3.62×10^{-8}, 1.04×10^{-7}]	-7.23 ± 0.094	0.990
Tolazoline	1.20×10^{-7}	[5.93×10^{-8}, 2.77×10^{-7}]	-6.92 ± 0.129	0.982
Clonidine	5.45×10^{-7}	[2.17×10^{-7}, 2.01×10^{-6}]	-6.26 ± 0.159	0.958
Naphazoline	1.29×10^{-10}	[5.57×10^{-11}, 2.95×10^{-10}]	-9.89 ± 0.146	0.976

Table S5. IC_{50} and $LogIC_{50}$ values for inhibition of OA-induced cAMP response by various antagonists in HEK 293T cells expressing DcOctβ1R.

Antagonists	IC_{50} (M)	95% CI for IC_{50} (M)	Log IC_{50} (mean ± SEM)	Fitting R^2
Mianserin	1.06×10^{-5}	[5.85×10^{-6}, 1.89×10^{-5}]	-4.97 ± 0.103	0.987
Epinastine	3.80×10^{-8}	[2.50×10^{-8}, 5.76×10^{-8}]	-7.418 ± 0.076	0.990
Chlorpromazine	2.56×10^{-6}	[6.27×10^{-7}, 9.27×10^{-6}]	-5.59 ± 0.229	0.949
Metoclopramide	4.90×10^{-6}	[2.68×10^{-6}, 8.65×10^{-6}]	-5.31 ± 0.102	0.985

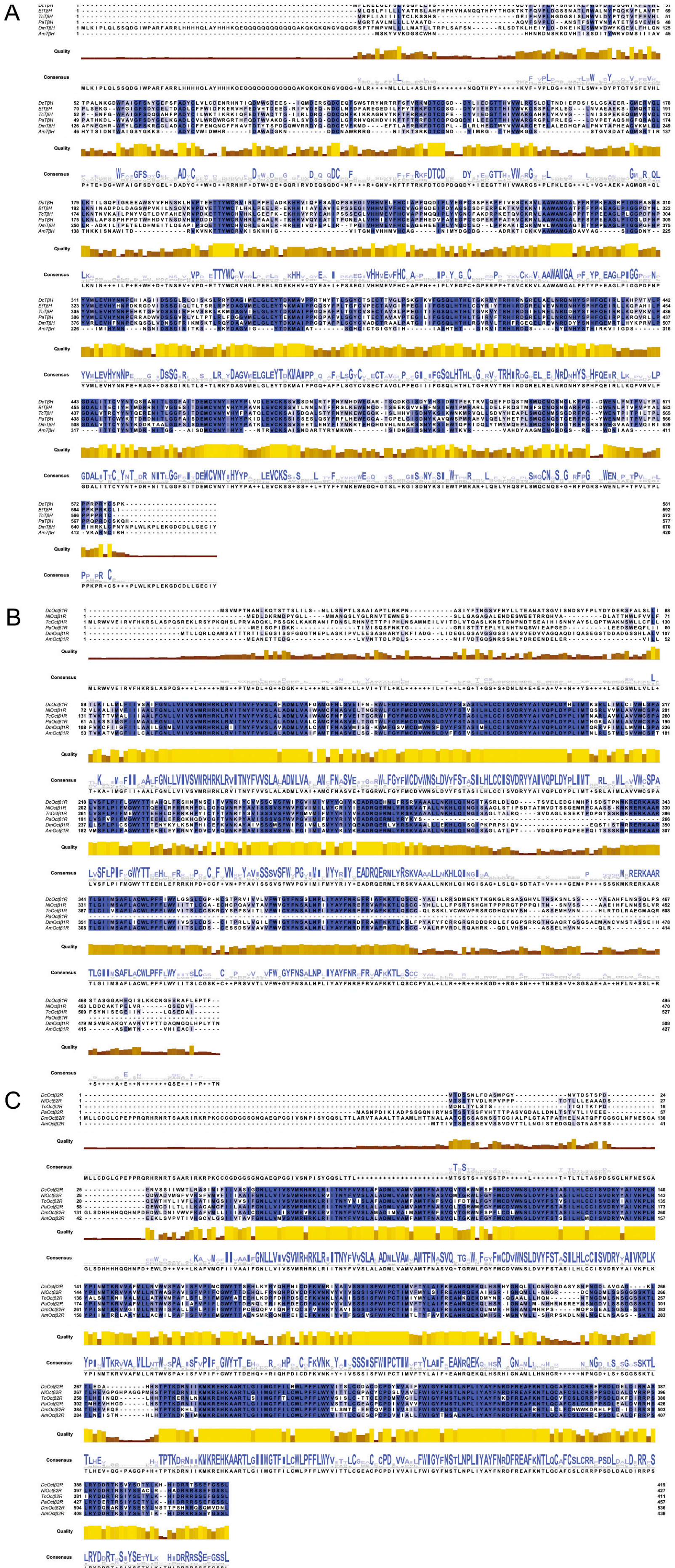
A
B
C
Quality
Consensus

Figure S1. Amino acid sequence alignment of *DcTβH* (A), *DcOctβ1R* (B) and *DcOctβ2R* (C) with orthologous genes. The numbers on the left and right indicate the position of the nucleotides and amino acids. Dashes are gaps that were introduced for alignment. The identical amino acid is colored letter showed in consensus. The quality of alignment is showed by yellow column. The shaded sequences highlight the identity level of amino acids between the orthologous sequences. Identical amino acids are highlighted in dark blue and conserved amino acids are on light blue, as determined using the 50% majority rules. The accession numbers used in the sequence alignment were listed in Table S2.

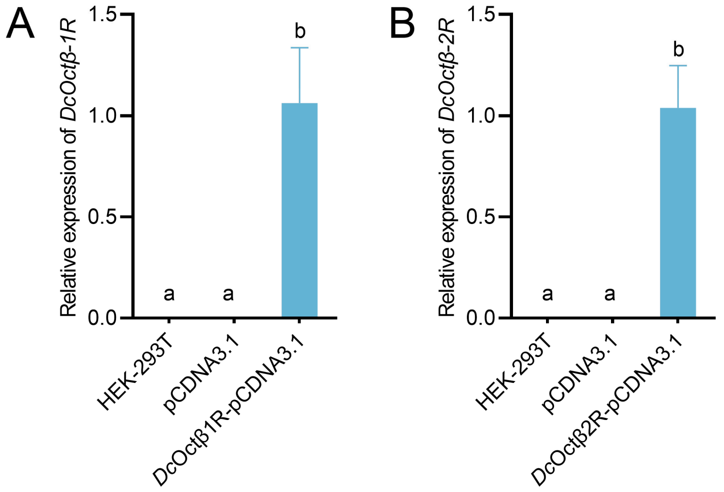

Figure S2. The relative expression of *Dc*Octβ1R (A) and *Dc*Octβ2R (B) in HEK-293T cells. Different letters above the bars indicate significant differences at P < 0.05 (Tukey's post hoc test)Data are the means of three replicates ± SEM.

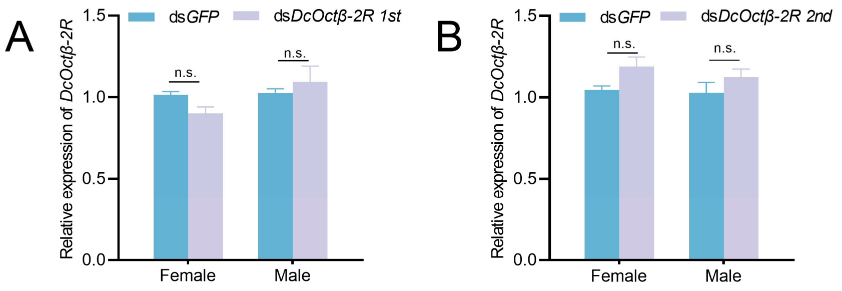

Figure S3. Relative expression of *DcOctβ2R* after ds*DcOctβ2R* treatment for 24h. "n.s." denotes no significant difference ($P > 0.05$, Student's t test). Data are presented as means of three replicates ±

SEM.
